# QuickStats

**Published:** 2015-06-05

**Authors:** FROM THE NATIONAL CENTER FOR HEALTH STATISTICS

**Figure:**
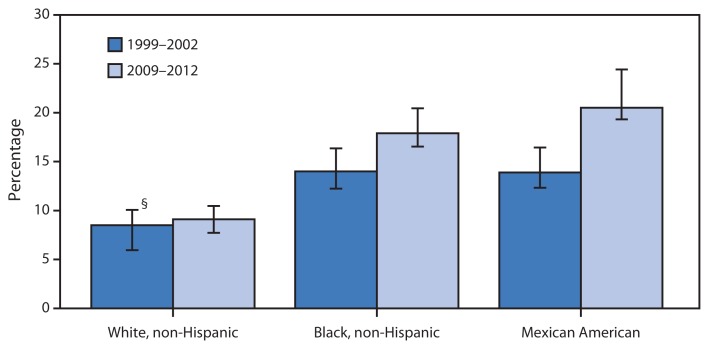
Age-Adjusted* Percentage of Adults Aged ≥20 Years with Diabetes,^†^ by Race and Hispanic Ethnicity — National Health and Nutrition Examination Survey, United States, 1999–2002 and 2009–2012 * Estimates are age-adjusted; pregnant women are excluded. ^†^ Diabetes is defined as measured fasting plasma glucose of at least 126 mg/dL, measured hemoglobin A1c of at least 6.5, or having been diagnosed by a physician. ^§^ 95% confidence interval.

From 1999–2002 to 2009–2012, the prevalence of diabetes increased for non-Hispanic black and Mexican American adults, but remained stable for non-Hispanic white adults, increasing the disparity with the two minority populations. In 1999–2002, the prevalence of diabetes among non-Hispanic black (14.0%) and Mexican American (13.9%) adults aged ≥20 years was 1.6 times the prevalence among non-Hispanic white adults (8.5%). By 2009–2012, diabetes prevalence among Mexican American adults (20.5%) had increased to more than twice the prevalence among non-Hispanic white adults (9.1%); among non-Hispanic black adults (17.9%), the prevalence had increased to nearly twice that among non-Hispanic white adults.

**Source:** Health, United States, 2014: with special feature on adults aged 55–64. Table 44. Available at http://www.cdc.gov/nchs/hus.htm.

**Reported by:** Sheila J. Franco, sfranco@cdc.gov, 301-458-4331; Shilpa Bengeri.

